# Present and future perspectives in early diagnosis and monitoring for progressive fibrosing interstitial lung diseases

**DOI:** 10.3389/fmed.2023.1114722

**Published:** 2023-02-15

**Authors:** Stefan Cristian Stanel, Pilar Rivera-Ortega

**Affiliations:** ^1^Interstitial Lung Disease (ILD) Unit, North West Lung Centre, Wythenshawe Hospital, Manchester University NHS Foundation Trust, Wythenshawe, United Kingdom; ^2^Faculty of Biology, Medicine and Health, University of Manchester, Manchester, United Kingdom

**Keywords:** progressive fibrosing interstitial lung disease, progressive pulmonary fibrosis, interstitial lung disease, idiopathic pulmonary fibrosis, PF-ILD, PPF

## Abstract

Progressive fibrosing interstitial lung diseases (PF-ILDs) represent a group of conditions of both known and unknown origin which continue to worsen despite standard treatments, leading to respiratory failure and early mortality. Given the potential to slow down progression by initiating antifibrotic therapies where appropriate, there is ample opportunity to implement innovative strategies for early diagnosis and monitoring with the goal of improving clinical outcomes. Early diagnosis can be facilitated by standardizing ILD multidisciplinary team (MDT) discussions, implementing machine learning algorithms for chest computed-tomography quantitative analysis and novel magnetic-resonance imaging techniques, as well as measuring blood biomarker signatures and genetic testing for telomere length and identification of deleterious mutations in telomere-related genes and other single-nucleotide polymorphisms (SNPs) linked to pulmonary fibrosis such as rs35705950 in the MUC5B promoter region. Assessing disease progression in the post COVID-19 era also led to a number of advances in home monitoring using digitally-enabled home spirometers, pulse oximeters and other wearable devices. While validation for many of these innovations is still in progress, significant changes to current clinical practice for PF-ILDs can be expected in the near future.

## 1. Introduction

Within the complex landscape of interstitial lung diseases (ILDs), a widely studied disease and a major new concept have emerged with the publication of the updated 2022 ATS/ERS/JRS/ALAT clinical practice guideline: progressive pulmonary fibrosis (PPF). (1) While idiopathic pulmonary fibrosis (IPF) is a diagnosis of exclusion, with an unknown etiology and a grim prognosis rivaling most cancers, (2) PPF includes a multitude of ILDs, of both known and unknown origin, that share a progressive disease behavior.

As there is currently approved antifibrotic therapy for progressive fibrosing interstitial lung diseases (PF-ILD)—nintedanib, there is some confusion among clinicians as to what definition to use for establishing progression. The PPF criteria (1), as well as the nintedanib for PF-ILD INBUILD trial inclusion criteria (3) are most often used. Other definitions exist, based on two other studies (pirfenidone in unclassifiable ILD [uILD] and RELIEF) as well as criteria proposed by Cottin et al. (4)—all definitions are detailed in Table 1.

**Table 1:** Summary of several definitions which could be used in clinical practice to define progression of fibrosing interstitial lung diseases.

Definition of progression in fibrosing ILDs	
PPF criteria (1)	Two of the following criteria met within the last year without an alternative explanation: 1. Worsening respiratory symptoms
	2. Lung function decline within 1 year of follow-up—either of absolute decline in forced vital capacity (FVC) ≥ 5% predicted, or absolute decline in DLCO (corrected for hemoglobin) ≥ 10% predicted
	3. Radiological progression—defined in the 2022 ATS/ERS/JRS/ALAT guideline (1) “as one or more of the following: a. Increased extent or severity of traction bronchiectasis and bronchioloectasis b. New ground-glass opacity with traction bronchiectasis c. New fine reticulation d. Increased extent or increased coarseness of reticular abnormality e. New or increased honeycombing f. Increased lobar volume loss”
INBUILD criteria (3)	At least one of the following criteria met within 24 months, despite standard treatment with a therapy other than nintedanib or pirfenidone: 1. Relative decline in the FVC ≥ 10% of the predicted value
	2. Relative decline in the FVC > 5% to <10% of the predicted value **plus** worsening respiratory symptoms **or** increased fibrosis on chest HRCT
	3. Worsening respiratory symptoms **and** increased fibrosis on chest HRCT
Pirfenidone in uILD criteria (5)	Either of the following criteria met within the previous 6 months: 1. Absolute decline in FVC > 5% of percent predicted **or** 2. Worsening respiratory symptoms not explained by cardiac, vascular, pulmonary (except ILD) or other causes
RELIEF criteria (6)	Within 6–24 months prior to inclusion, annualized (absolute) decline in FVC ≥ 5%
Cottin et al. proposed criteria (7)	Either of the following criteria met within a 24 month period: 1. Absolute decline in FVC ≥ 10% 2. Absolute decline in DLCO ≥ 15% 3. Worsening respiratory symptoms 4. Worsening radiological appearance accompanied by a ≥ 5 to < 10% relative decrease in FVC

FVC = forced vital capacity, DLCO = diffusing capacity of the lung for carbon monoxide. HRCT = high-resolution computed tomography, uILD = unclassifiable interstitial lung disease.

Choosing how to document progression has practical implications in obtaining reimbursement for nintedanib, where specific local requirements may need to be met.

Assuming that non-IPF ILDs can behave similarly to IPF and meet criteria for PF-ILD in up to 32% of cases (8), we aimed to summarize and discuss some of the emerging trends in the diagnosis and monitoring of this varied group of conditions, occasionally drawing parallels to IPF as the prototype of progressive fibrotic ILD. The clinical characteristics of the various non-IPF ILDs that could be included under the PF-ILD umbrella have been reviewed previously (9).

## 2. Current and future directions

### 2.1. The importance of multidisciplinary team discussion

Multidisciplinary team (MDT) consensus diagnosis for ILD as a gold standard has been suggested; however, the practice of organizing these meetings varies greatly around the world. Before the COVID-19 pandemic, a survey performed across 64 countries by the Respiratory Effectiveness Group (REG) revealed that 76% of centers held formal MDT meetings and the majority (80%) were face-to-face (10). This survey is currently being repeated to better understand how teleworking and the pandemic have influenced MDT practices. It is not clear how MDT discussions are organized in developing countries and what are the opportunities for improvement.

Requirements including a quiet setting with a video projection system, at least one radiologist present, access to high-quality HRCT of the chest, and a standardized template summarizing patient data were deemed essential components of the MDT meeting in a recent Delphi survey of ILD experts (11). Diagnosis of connective-tissue disease-associated ILD (CTD-ILD) would require the presence of a rheumatologist or immunologist for the MDT discussion. However, just over a third of all centers in the REG survey routinely involved these specialists in the discussion (10).

There are benefits to holding MDT meetings, including increased diagnostic confidence and inter-observer agreement, and lower rates of unclassifiable ILD diagnoses. The meetings also provide a forum for discussion and sharing knowledge and experience (12). As we emerge from the COVID-19 pandemic, virtual MDT discussions have brought new opportunities, especially by increasing the number of attendees (including trainees and non-specialist physicians). However, virtual meetings can be less accessible in resource-poor areas, less focused, and prone to “technical” difficulties. Preserving patient confidentiality may also prove difficult in virtual settings (13).

The future of ILD MDT discussion is likely going to include genetic testing data and input from relevant specialists (i.e., clinical genetics, lung transplant physicians) due to recent discoveries of accelerated progression and worse responses to immunosuppression in patients with familial forms of ILD or sporadic cases with a genetic component (e.g., telomere dysfunction). There is significant support from clinicians, as well as patients and their relatives for genetic testing (14).

In our experience, MDT consensus also builds diagnostic confidence from a patient perspective and provides reassurance that an entire ILD team is involved in care provision. In our center, ILD specialist nurses and pharmacists also regularly attend MDT discussions to provide their own unique input regarding potential tolerability and interactions when considering treatments for PF-ILD.

### 2.2. Imaging and CT quantitative analysis

Early attempts at defining imaging biomarkers for ILD progression were focused on chest CT patterns present at diagnosis. The finding of a usual interstitial pneumonia (UIP) pattern on chest CT in hypersensitivity pneumonitis (HP) was associated with a similar rate of lung function decline in PF-ILD compared to IPF. Similarly, in rheumatoid arthritis associated ILD (RA-ILD), UIP was identified as a major predictor of decline (15). However, there can be significant inter- and intra-observer variability for visual radiological evaluation, especially in non-UIP pattern fibrosis.

Some progress has been made in improving the diagnostic and monitoring accuracy of ILDs using artificial intelligence. The Computer Aided Lung Informatics for Pathology Evaluation and Rating (CALIPER) program seemed to be able to differentiate between IPF and CTD-ILD, showing differences in analysis of peripheral volume of reticulation (greater in IPF versus CTD) and vascular-related structure (VRS) volume (greater in IPF versus CTD) (16). In IPF patients, CALIPER quantification scores for ILD (ILD%) and pulmonary vascular-related structures (PVRS%) were shown to correlate with forced vital capacity (FVC) at baseline evaluation and during disease progression, with faster increases in scores in patients who were not treated with antifibrotics (17). For non-IPF ILDs, similar findings are starting to emerge, with reticulation and traction bronchiectasis scores (QLF) predicting survival in RA-ILD (18) and convolutional neural network approaches in HP showing correlation with lung function parameters (19).

There are however inherent challenges. Machine learning algorithms require “training” using quality data and there are issues with validating the accuracy of the results. Furthermore, most studies have been retrospective, with not enough longitudinal data to estimate whether automated quantitative CT analysis will indeed positively impact clinical outcomes (20). A recent systematic review confirmed the need to increase diagnostic accuracy and gather prospective data (21). The PREDICT-ILD study will hopefully shed some light on the use of CT quantification for predicting lung function trajectories in fibrotic ILDs and correlate scores with genetic predisposition and markers of endothelial damage (NCT05609201).

Although not validated for routine clinical practice, a promising area of investigation is the use of magnetic-resonance imaging (MRI) based techniques for the evaluation of ILDs of different etiologies. Conventional MRI has inherent difficulties in imaging the lung parenchyma; however, techniques such as ultrashort echo time or dynamic contrast-enhanced MRI can be helpful for imaging the lung vasculature. Inhaled hyperpolarized ^129^Xenon gas MRI can provide a functional assessment of alveolar-capillary diffusion as well as ventilation and intra-acinar gas diffusion (22). So far, ^129^Xe ventilation or oxygen enhanced-MRI biomarkers were not able to discriminate between the different types of ILD in one small study (23). However, dynamic contrast-enhanced perfusion MRI seemed to correlate with pulmonary vascular disease progression in IPF (24) which would be a relevant biomarker in the monitoring of PF-ILD. Further assessment of ^129^Xe MRI is also underway as part of the UKILD consortium (in the evaluation of post COVID-ILD patients) (25)

### 2.3. Home monitoring for PF-ILD

The COVID-19 pandemic has catalyzed the development of home monitoring strategies for fibrotic lung disease, as many centers struggled to maintain face-to-face patient encounters and availability for hospital-based lung function testing became severely reduced. The severity of lung function impairment has been demonstrated to be one of the most important predictors of worse outcomes in non-IPF PF-ILDs (15).

Home spirometry involves providing patients with a device that normally connects to their smartphone *via* Bluetooth®, allowing real-time uploading of results to a patient portal (which may also be accessible to the physician). Normally, initial training and device setup are done in clinic, the patient then being asked to perform home spirometry according to a set schedule (i.e., once daily, once weekly). Instructional videos are sometimes available, and some platforms allow automated reminders to be set up with the goal of increasing adherence.

A systematic review has shown that patient adherence to home spirometry was satisfactory (> 75%) and values measured at home correlated significantly with those measured in-hospital (26) Interestingly, the variability in home-measured FVC values may actually be an independent predictor for fibrotic ILD progression (27) Increasing adherence can be achieved by setting up automated email reminders when a measurement is not performed when expected (28), providing comprehensive initial and refresher training to patients, or using a spirometry schedule which is more acceptable (rather than daily measurements) (29). The optimal timing and frequency of testing to account for diurnal variation has not yet been established (30).

Home spirometry allows for trends in lung function decline to be generated, which is of great importance in monitoring and increasing diagnostic accuracy for PF-ILD. Additionally, as many patients could not be seen often enough during the pandemic, a role emerged for home spirometry to aid with early diagnosis of acute exacerbations of ILD (26).

While useful in a clinical setting, there are accuracy limitations to incorporating home spirometry FVC decline as a primary endpoint in clinical trials for PF-ILD, as demonstrated in a phase 2 study of pirfenidone for unclassifiable PF-ILD (5). In this study, estimating the rate of FVC decline proved difficult due to technical difficulties with the device and implausible measurements. Similar issues were encountered in two other studies aiming to describe ILD disease behavior using home spirometry (STARLINER and STARMAP) (29). Despite the limitations, high patient satisfaction with home spirometry monitoring has been reported (30, 31).

Ambulatory pulse oximetry coupled with activity monitoring has been used to provide continuous data on peripheral oxygen saturation (SpO2) to help optimize long-term oxygen treatment (32). Consumer-level activity trackers (e.g., Fitbit, San Francisco, CA, United States) can record multiple parameters including step counts, heart rate, heart rate variability, SpO2, and skin temperature. Data from a small study in sarcoidosis reported an improvement in exercise performance in patients wearing an activity tracker compared to controls (33). Perceived positive effects may drive many patients to self-initiate activity monitoring using wearables. Integrating these data into clinical care may prove difficult, due to variability in measurements and inability to deconstruct proprietary algorithms which present recorded data in a consumer-friendly format. It is unknown which parameters will yield the greatest clinical benefit, however this area of research is promising (30).

Cough-frequency monitoring can provide objective symptomatic monitoring for PF-ILD patients to aid in treatment decisions (i.e., prescribing cough suppressants). Existing devices such as the VitaloJAK (Vitalograph, Buckingham, United Kingdom) or the Leicester Cough Monitor (University Hospital Leicester, Leicester, United Kingdom) have been mostly used in clinical trials, and there may be limitations to their use in outpatient settings (34). Methods which involve cough monitoring *via* smartphone applications are currently being developed (35). The main drawback of implementing cough monitoring at scale is the need to protect patient privacy, as sound needs to be recorded and analyzed.

### 2.4. Blood biomarkers

Much of the work regarding serum and plasma biomarkers in ILD has so far focused on IPF. Since there is overlap between IPF and non-IPF PF-ILDs with respect to molecular pathways, emerging data suggest that there is also overlap in the biomarkers of interest (36). While it is unlikely that a single biomarker would explain the full spectrum of PF-ILD, combining several markers into “signatures” can enhance their clinical utility.

In IPF, a progression index based on 4 biomarkers (osteopontin—OPN, matrix metallopeptidase-7—MMP-7, intercellular adhesion molecule-1—ICAM1, and periostin—POSTN) was found to be superior to the clinical GAP score (gender, age, and lung physiology) in predicting progression at 12 months (37). A combination of MMP-7, pulmonary and activation-regulated chemokine (PARC), and surfactant-protein D (SP-D) increased the predictive value of clinical features, positive rheumatoid factor, and anti-cyclic citrullinated peptide antibodies for RA-ILD (38). Bowman et al. also recently used a proteomic approach to identify 17 biomarkers for PF-ILD which had consistent associations across different ILDs and chest HRCT imaging patterns. This data support a shared pathophysiology across the PF-ILD spectrum and paves the way for using a proteomic signature for defining progressive fibrosis. The ITGB6 marker (which represents the β6 subunit of integrin αvβ6, a critical activator of TGF-β) was found to have the strongest association with progressive fibrosis (39).

Prospective data on the use of biomarkers in influencing clinical outcomes are still lacking. One of the main aims of the INJUSTIS study (currently recruiting) is to obtain longitudinal data on biomarkers which predict progressive fibrosis in non-IPF patients (NCT03670576).

### 2.5. Genetic biomarkers

An ever-increasing body of evidence suggests that the development of ILD is rooted in genetic factors. The study of familial cases has yielded a number of deleterious mutations in several telomere-related genes (TRGs), which lead to premature telomere attrition. These include telomerase reverse transcriptase (TERT), telomerase RNA component (TERC), dyskerin (DKC1), regulator of telomere elongation helicase (RTEL1), poly(A)-specific ribonuclease (PARN), surfactant protein C (SFTPC) and A2 (SFTPA2), and the shelterin complex (also known as the telosome, and consisting of TRF1, TRF2, RAP1, TIN2, POT1, and TPP1) (40, 41). Telomere dysfunction has been implicated in all forms of ILD, of which many have a progressive fibrosing phenotype (42). There is significant overlap between IPF as a prototype of progressive fibrotic lung disease (IPF) and other PF-ILDs (43)

Using genome-wide association studies (GWASs), several groups found a strong association between the rs35705950 single-nucleotide polymorphism (SNP) in the MUC5B promoter and IPF and interstitial lung abnormalities (ILAs) (44–46). The MUC5B variant was also associated with the risk of developing a UIP pattern on chest CT scanning in HP and RA-ILD which confers the highest risk of fibrosis progression (47, 48). In HP patients, the MUC5B high-risk polymorphism was found in approximately a quarter of patients compared to 10% in the general population (48). Research into novel causes fibrosis also revealed correlations suggesting an overlap between genetic predisposition for fibrotic conditions (i.e., IPF) and severe COVID-19 (49).

From a clinical perspective, although the overall phenotype may not be different in familial versus sporadic ILD cases, disease onset tends to be early. Within the same family, heterogeneity of ILD diagnosis may be possible, which is not fully understood, but may relate to an interplay with environmental and developmental factors (50). Sporadic IPF cases with an early onset (age < 60 years) had a higher likelihood of having telomere shortening, notably if they also featured immunological or hematological abnormalities (51). Telomere attrition was found in up to a quarter of patients with sporadic IPF and up to half of those with familial pulmonary fibrosis (52).

Heterozygous mutations in TRGs were associated with a uniformly progressive fibrotic phenotype (regardless of ILD diagnosis) and patients had a mean annual decline in FVC of 300 ML, which is more rapid than the 130–210 ML/year FVC loss seen in placebo arms of IPF clinical trials (53).

Progressive fibrosing interstitial lung disease treatment often involves immunosuppressant therapy and clinicians need to carefully monitor patients with telomere shortening due to a greater risk of developing treatment-related side effects (as seen in IPF with the PANTHER-IPF trial and fibrotic HP) (54, 55). Complications and worse outcomes after lung transplantation were noted for patients with short telomeres (54). However, in a Spanish cohort of 20 patients with fibrotic ILD who underwent lung transplantation (12 with and 8 without telomere shortening), post-transplant 1-year survival was > 80% regardless of telomere dysfunction, with improvement in the quality of life and manageable complications (56). Loss of clinical efficacy of immunosuppression is also suggested by findings of mycophenolate treatment only leading to improvement in fibrotic HP patients who had normal leukocyte telomere length (57).

Telomere dysfunction may confer a higher likelihood of negative responses to environmental insults (such as exposure to particulate matter) although such research is mired with difficulties in determining correlations without confounding (58).

Taken together, these findings implicate a definite role for genetic predisposition in the development of PF-ILD. In practical terms, this means that clinicians should actively ask about family history and identify clinical features of telomere dysfunction when diagnosing and treating PF-ILD; and to refer at-risk individuals for genetic testing as appropriate.

## 3. Discussion

**Figure 1:**
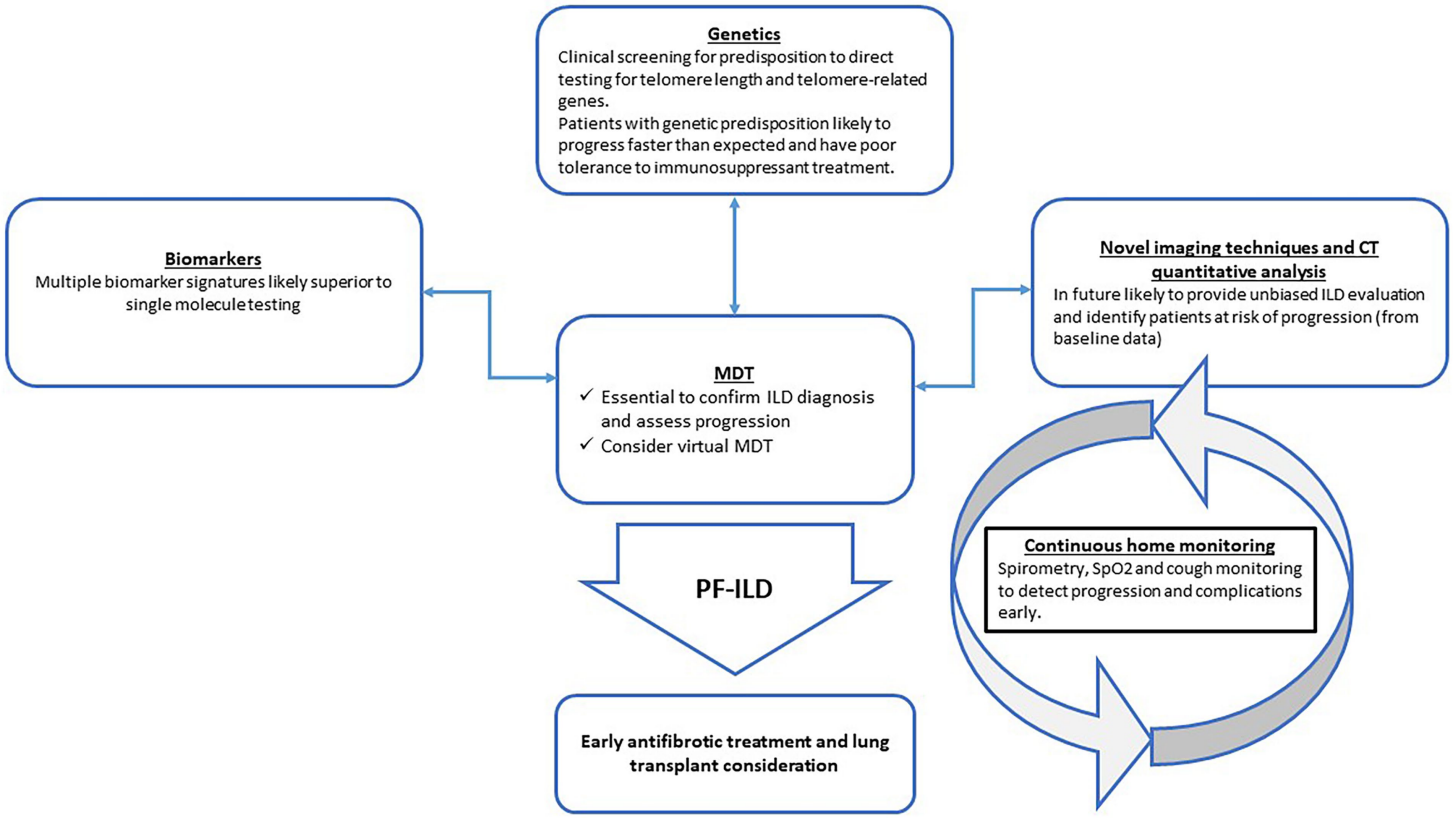
Proposed integration of novel tools to improve early diagnosis and monitoring of progressive-fibrosing interstitial lung diseases (PF-ILDs) in the clinical setting. MDT = multi-disciplinary team; CT = computed tomography; SpO2 = peripheral oxygen saturation.

There has been significant progress in improving the accuracy of PF-ILD diagnosis and developing novel monitoring strategies. Early identification of patients at risk of PF-ILD by deconvoluting the complex landscape of genetic predisposition and other biomarkers holds the promise of avoiding inherent delays in diagnosis, which currently requires documented evidence of decline in symptoms, lung function or imaging parameters over 12–24 months (Figure 1).

The INBUILD trial showed that antifibrotic treatment with nintedanib versus placebo in PF-ILD reduced the annual adjusted rate of FVC decline from approximately 180 ML to 80 ML, with an even greater difference seen in those with a UIP pattern on imaging (3), leading to a conditional recommendation for nintedanib in PF-ILD (1). Early initiation of treatment is essential.

Technological approaches are likely to become a routine part of PF-ILD monitoring in the near future and it is important to become familiarized with the various home spirometry, pulse oximetry, and activity monitoring platforms. Although further validation of these devices is required, many patients are already using them to gain personal health insights and clinicians should be ready to integrate this data into routine follow-up.

Machine learning tools are likely to help reduce inter- and intra-observer variability of imaging data, which will allow for more accurate ILD diagnosis and identifying those patients most at risk of progression.

Finally, in our opinion, large improvements in the care of PF-ILD patients could be obtained by simple adjustments to clinical practice, such as encouraging a standardized approach to ILD MDT discussion involving expert opinion from specialist centers (which can be done virtually) and by routinely asking about family history to uncover at-risk relatives of ILD patients early.

## Data availability statement

The original contributions presented in the study are included in the article/supplementary material, further inquiries can be directed to the corresponding author.

## Author contributions

SS and PR-O have both contributed equally to the conceptualization, literature review, drafting of the manuscript and approved the final version.

## Conflict of interest

The authors declare that the research was conducted in the absence of any commercial or financial relationships that could be construed as a potential conflict of interest.

## Publisher’s note

All claims expressed in this article are solely those of the authors and do not necessarily represent those of their affiliated organizations, or those of the publisher, the editors and the reviewers. Any product that may be evaluated in this article, or claim that may be made by its manufacturer, is not guaranteed or endorsed by the publisher.
